# Using artificial intelligence tools to automate data extraction for living evidence syntheses

**DOI:** 10.1371/journal.pone.0320151

**Published:** 2025-04-03

**Authors:** Evan Mitchell, Elisha B. Are, Caroline Colijn, David J. D. Earn

**Affiliations:** 1 Department of Mathematics and Statistics, McMaster University, Hamilton, ON,Canada; 2 Department of Mathematics, Simon Fraser University, Burnaby, BC,Canada; Ascension Sacred Heart Hospital Pensacola, UNITEDSTATES OF AMERICA

## Abstract

Living evidence synthesis (LES) involves repeatedly updating a systematic review or meta-analysis at regular intervals to incorporate new evidence into the summary results. It requires a considerable amount of human time investment in the article search, collection, and data extraction phases. Tools exist to automate the retrieval of relevant journal articles, but pulling data out of those articles is currently still a manual process. In this article, we present a proof-of-concept Python program that leverages artificial intelligence (AI) tools (specifically, ChatGPT) to parse a batch of journal articles and extract relevant results, greatly reducing the human time investment in this action without compromising on accuracy. Our program is tested on a set of journal articles that estimate the mean incubation period for COVID-19, an epidemiological parameter of importance for mathematical modelling. We also discuss important limitations related to the total amount of information and rate at which that information can be sent to the AI engine. This work contributes to the ongoing discussion about the use of AI and the role such tools can have in scientific research.

## Introduction

Systematic reviews and meta-analyses are key tools used across many disciplines for synthesizing large bodies of research. They involve critically evaluating research methodologies and employ statistical techniques to combine study results into summary conclusions. However, such reviews have a limited shelf life. As more studies are published, the findings of meta-analytic work become outdated. In some extreme cases, like during the COVID-19 pandemic when an estimated 1.5 million new research articles were added to the global literature base in 2020 alone [1], systematic reviews can be outdated as soon as they are published.

This important limitation has lead to the rise of so-called “living” evidence synthesis (LES) [2]. Essentially, this is a systematic review that is repeated at regular intervals to incorporate any new evidence that has been published since the last update. While this keeps the summary conclusions relevant, it also requires a significant human time investment. With each update, researchers need to manually search for new studies, evaluate their relevance and research methodologies, extract study metadata and results, and incorporate those results into the previous review findings.

Machine learning techniques have helped to considerably reduce this manual time investment [3–5]. Researchers are now able to use natural language processing to scan article titles and abstracts to create lists of relevant studies [6–8], remove duplicate studies in these compiled lists [9], and train machine learning models to extract article metadata and evaluate risk of bias from abstracts in clinical studies that follow a very specific structure [10,11]. Comprehensive platforms for systematic reviews, like Thalia [12], SyRF [13], or SOLES [14], exist to apply these machine learning techniques and organize all stages of a meta-analysis. However, at the time of writing, the data extraction phase is still done manually across all of these platforms. Researchers must spend considerable time going through their collected articles to record relevant study results. Our goal in this article is to show that the recent advent of artificial intelligence (AI) tools like ChatGPT can be leveraged to partially automate this time-intensive process. We introduce a Python script that can be used to interface with ChatGPT’s application programming interface (API) and extract study results, and discuss associated performance and limitations. Our focus is on applying this program to retrieving estimates of parameters used in mathematical models of infectious disease spread. In particular, we test the performance of our AI program on research articles estimating the average incubation period (the time from infection to the onset of symptoms) for COVID-19 and compare this to manual extraction of these same data.

## Methods

Our AI script, hereafter referred to by the acronym AI-LES (Artificial Intelligence - Living Evidence Synthesis), is a Python script designed to interface with ChatGPT to ask a series of questions about a journal article. We test AI-LES on a set of 94 journal articles that were manually screened for inclusion in a previous meta-analysis on the incubation period of COVID-19 [15]; a complete list of these articles is found in PDF format in S1 Appendix or as a BibTeX file in S2 Appendix. For each article, we ask the following questions:

1. What is the estimate for the incubation period from this paper?
2. Is this an estimate of the mean or median incubation period?
3. What is the 95% confidence interval, range, or IQR for the incubation period?
4. Is this a confidence interval, a range, or an IQR?
5. What is the standard deviation or standard error of the incubation period?
6. Is this a standard deviation or a standard error?
7. Does this paper present incubation periods for different subgroups of individuals? If no, please return a 0. If yes, please list the subgroups.

During testing, it was found that AI-LES would return an estimate of the median incubation period if no estimate of the mean was given. Question 2 is provided to disambiguate whether it is returning an estimate of the mean or the median. Questions 4 and 6 are similarly present to disambiguate the values returned by AI-LES. Finally, some research articles provide estimates for the incubation period within multiple subgroups (e.g., ethnic groups, gender groups, or geographical regions); Question 7 is used to flag such articles for closer inspection by a researcher.

An important consideration when working with any type of large language model (LLM) like ChatGPT is the effect of hallucinations, whereby the LLM returns false information. Two techniques are employed here to reduce the likelihood of hallucinations. First, the prompts are designed with a chain-of-thought strategy in mind [16]. Complex prompts are replaced by a sequence of shorter, more direct prompts that lead to improved accuracy. Second, by providing a reference text on which the LLM can base its answers, we are using a retrieval augmented generation (RAG) approach, which has been shown to help reduce hallucinations [17].

**Fig 1:**
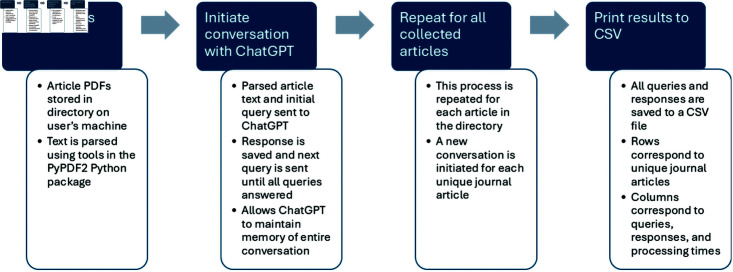
Flowchart describing the AI-LES program.

Our AI-LES program is implemented in Python using the openai library to interface with ChatGPT. A copy of the code can be found in PDF format in S3 Appendix or as a Python code file in S4 Appendix. Fig 1 shows a flowchart describing the main steps involved in this program.

To interface with the ChatGPT API, users first need their own individual API key. This can be received by creating an account with the company that owns ChatGPT, OpenAI (https://openai.com/). This API key needs to be set at the start of the script.

AI-LES starts by creating a function that takes all previous queries passed to the API and all responses received from the API, combined with the current query, and passes them to the API. This allows the API to “remember” the queries and responses up to this point, effectively simulating an ongoing conversation between the user and ChatGPT’s AI engine. ChatGPT has multiple models available for use with various functionalities associated with each. Here, we use the gpt-3.5-turbo-0125 model which is primarily useful for interpreting natural language.

For each journal article, we use Python’s PyPDF2 package to read in the article PDF file. An initial query is combined with this parsed text and sent to ChatGPT to get a response. We then loop through the remainder of our queries, using our previously defined function, and ask for a response from ChatGPT. The results are then printed to a CSV file where each row corresponds to a single journal article and each column corresponds to a single query about that article.

ChatGPT’s API limits the number of requests that can be sent per minute. To account for this, AI-LES implements an exponential backoff algorithm. If a rate limit error is returned, the code pauses for a variable amount of time. The pause starts at 2 seconds and doubles if a rate limit error is still hit; this timer then restarts each time AI-LES moves onto a new journal article. Implementing the pause in this way substantially reduces the total wait time in executing the code.

## Results

**Fig 2:**
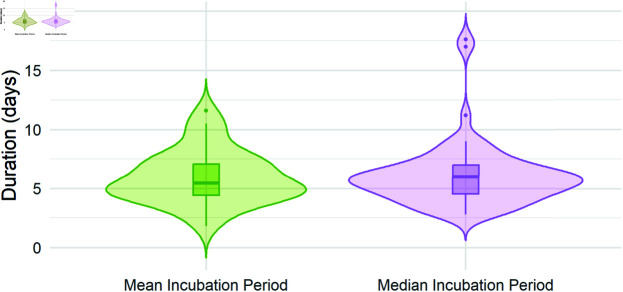
Violin plots showing the estimates of the mean (green) and median (purple) incubation period for COVID-19. Of the 94 sample studies, 32 provide an estimate of the mean and 62 provide an estimate of the median. Within each violin is a box plot showing the 25th, 50th, and 75th quartiles along with any outliers.

Our primary interest is in the degree to which our AI program successfully extracts desired results from these journal articles. We looked at both accuracy, meaning whether the correct values were returned as stated in each article, and reliability, meaning whether the same values were returned each time the program is run on the same article. The performance of the program is similar across each of the seven questions discussed above. In cases where the journal article clearly presents the statistics of interest, AI-LES accurately and reliably returns these quantities 100% of the time. In such cases, it is also able to distinguish between means and medians, confidence intervals and interquartile ranges, and standard deviations and standard errors, with 100% accuracy. AI-LES struggles in the same situations where a human researcher would struggle; namely, in cases where the information presented in the journal article is incomplete. For example, if an estimate of the incubation period is not given or if it is not clearly stated whether the estimate provided is of the mean or median incubation period, AI-LES may hallucinate a response. However, such occurrences are directly correlated with the clarity of the journal article under investigation and articles that do not clearly present findings are likely to be excluded *a priori* from LES reviews. Fig 2 shows the extracted estimates of the incubation period of COVID-19, grouped based on whether the article provides an estimate of the mean or the median incubation period.

**Fig 3:**
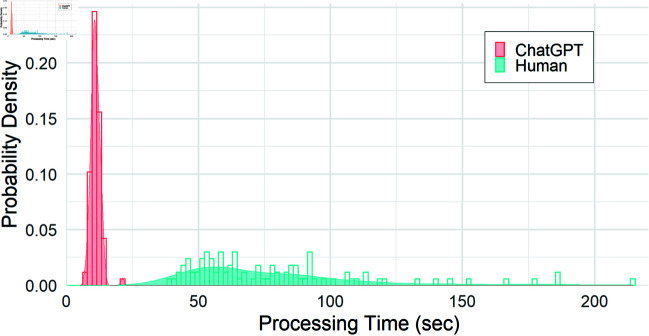
Distribution of processing times for the 94 sample articles. Red represents the AI script and blue represents manual data extraction. The mean of the ChatGPT distribution is 11.06 seconds with a standard deviation of 1.87 seconds. The mean of the Human distribution is 79.13 seconds with a standard deviation of 35.70 seconds.

An additional important metric is the time it takes to extract the results from the journal articles. Fig 3 shows the distribution of processing times, both human and AI, for the journal articles in our sample set. To process all 94 articles in our test sample took AI-LES a total of 76 minutes, averaging out to approximately 48 seconds per article. However, on average, only 11 seconds of this time is actually devoted to processing the article and retrieving results. The remainder is waiting time imposed by the ChatGPT API, as described in greater detail in the Discussion section. Human extraction took approximately 7 times longer with much greater variability in processing times. Articles that slow a human down considerably do not have the same effect on AI-LES, a finding that was further corroborated with a linear regression showing no significant correlation between AI-LES and human processing times.

Finally, we also evaluate AI-LES based on the monetary cost associated with using the ChatGPT API. ChatGPT charges based on the number of tokens of information used in both the query and response. Our sample of 94 articles had an associated total cost of $3.21 CAD. This works out to approximately $0.0341 CAD per article. These costs can be substantial for large sets of articles and could potentially exclude some users, especially in low-resource settings. However, these costs continue to decline over time with query costs being reduced by 50% and response costs reduced by 25% in January 2024 alone.

## Discussion and conclusion

In this article, we have sought to show how AI tools like ChatGPT can be leveraged to reduce human time investment in systematic reviews without compromising on accuracy. This is especially important for LES reviews where time investment is the main barrier to keeping such reviews up-to-date [14]. Reducing the human time investment also scales up the possible volume of information that can be summarized in such reviews; leveraging AI tools makes it feasible to incorporate thousands of journal articles in a review, something that would not be practical in a review done completely by hand. We have tested our AI-LES program on a set of papers estimating the incubation period of COVID-19, but such a program can be generalized to extract other parameters used in mathematical epidemiology with only small adjustments to the queries outlined above.

While the time savings of using AI can be considerable, there are also important technical limitations to which researchers must pay attention. These limitations concern the amount of information that can be sent or received in each request to ChatGPT’s API. ChatGPT measures information quantity in units of “tokens,” with one token being roughly equivalent to four characters in English language texts. Each version of ChatGPT’s model is associated with a different cap on the number of tokens that can make up a single request, imposing a limit on the number of queries that can be made of a single article or to the length of the article itself. There is also a rate limit in that only so many tokens can be sent or requested per minute. If that limit is exceeded, the AI model returns an error. This necessitated the implementation of a pause in our code that forces AI-LES to wait for a certain amount of time following a rate limit error before sending the next article to the AI engine. As mentioned in the Results section, this substantially increases the time that a researcher must currently wait for results from AI tools.

While our goal in this brief report is to present a proof-of-concept and generate discussion around the use of AI tools in LES reviews, there are some clear avenues for improving these types of tools. One direction would be to expand the scope of AI scripts like AI-LES beyond just results extraction to also pull information related to the quality of the study itself (e.g., statistical methodologies, mathematical modelling choices, the presence of computer code that can reproduce the study results). A second direction would be to move away from the limitations associated with commercially available AI engines like ChatGPT by building an in-house large language model (LLM). While beyond the scope of the current paper, a bespoke LLM trained on journal articles specifically estimating parameters of interest could be a valuable investment if the goal is to maintain a large database of many different epidemiological parameters.

A question that often arises in discussions related to the use of AI tools is whether those tools can completely replace human interaction. Given the current state and limitations of these tools, the answer for researchers conducting LES reviews appears to be “no.” Our AI-LES program shows that AI tools like ChatGPT are good at quickly retrieving data and results from journal articles. This opens the possibility of using these tools to supplement the LES lifecycle, reduce the human time spent on data extraction, and increase the volume of information included in LES protocols. Since AI is already used in the article collection phase of LES reviews, our results also suggest that LLMs could be used to automate the entire LES process by monitoring scientific databases for new articles and extracting relevant results. However, even if none of the previously mentioned limitations existed, AI tools as they are now still could not make judgement calls about the studies under investigation. They cannot evaluate the quality of data or the soundness of statistical methodologies or the appropriateness of mathematical models used in research. This still requires the input of a human researcher. Nevertheless, as AI tools continue to improve there does appear to be a clear role for them to fill in LES to complement human-led research.

## Supporting information

S1 Appendix References for program testing, pdf format. List of papers used for testing AI-LES program. (PDF)

S2 Appendix References for program testing, bib format. List of papers used for testing AI-LES program. (BIB)

S3 Appendix AI-LES source code, pdf format. Python script containing source code for AI-LES program. (PDF)

S4 Appendix AI-LES source code, py format. Python script containing source code for AI-LES program. (PY)
