# Supplementary material for: Using artificial intelligence tools to automate data extraction for living evidence syntheses
Source: PLoS One. 2025 Apr 3;20(4):e0320151. doi: 10.1371/journal.pone.0320151 (PMC11967977; doi:10.1371/journal.pone.0320151)
Supplement: S1 Appendix — (PDF) [file pone.0320151.s001.pdf]

Appendix: References for program testing

[1] T. Han *et al.*, "The epidemiological characteristics of cluster transmission of coronavirus disease 2019 (COVID-19): a multi-center study in Jiangsu Province," *Am J Transl Res*, vol. 12, no. 10, pp. 6434–6444, 2020.

[2] W. Zhu, M. Zhang, J. Pan, Y. Yao, and W. Wang, "Effects of prolonged incubation period and centralized quarantine on the COVID-19 outbreak in Shijiazhuang, China: a modeling study," *BMC Med*, vol. 19, no. 1, p. 308, Dec. 2021, doi: 10.1186/s12916-021-02178-z.

[3] J. Zhu *et al.*, "Challenges Caused by Imported Cases Abroad for the Prevention and Control of COVID-19 in China," *Front. Med.*, vol. 8, p. 573726, May 2021, doi: 10.3389/fmed.2021.573726.

[4] S. Zhao *et al.*, "Estimating the generation interval and inferring the latent period of COVID-19 from the contact tracing data," *Epidemics*, vol. 36, p. 100482, Sep. 2021, doi: 10.1016/j.epidem.2021.100482.

[5] H. Zhao *et al.*, "Transmission dynamics and successful control measures of SARS-CoV-2 in the mega-size city of Guangzhou, China," *Medicine*, vol. 100, no. 48, p. e27846, Dec. 2021, doi: 10.1097/MD.0000000000027846.

[6] T. Zhang *et al.*, "Estimation of Incubation Period and Serial Interval for SARS-CoV-2 in Jiangxi, China, and an Updated Meta-Analysis," *J Infect Dev Ctries*, vol. 15, no. 03, pp. 326–332, Mar. 2021, doi: 10.3855/jidc.14025.

[7] Q. Zhang, J. Zhu, C. Jia, S. Xu, T. Jiang, and S. Wang, "Epidemiology and Clinical Outcomes of COVID-19 Patients in Northwestern China Who Had a History of Exposure in Wuhan City: Departure Time-Originated Pinpoint Surveillance," *Front. Med.*, vol. 8, p. 582299, May 2021, doi: 10.3389/fmed.2021.582299.

[8] J. Zhang *et al.*, "Evolving epidemiology and transmission dynamics of coronavirus disease 2019 outside Hubei province, China: a descriptive and modelling study," *The Lancet Infectious Diseases*, vol. 20, no. 7, pp. 793–802, Jul. 2020, doi: 10.1016/S1473-3099(20)30230-9.

[9] H. Zhang *et al.*, "A Cluster Transmission of Coronavirus Disease 2019 and the Prevention and Control Measures in the Early Stage of the Epidemic in Xi'an, China, 2020," *Med Sci Monit*, vol. 27, Mar. 2021, doi: 10.12659/MSM.929701.

[10] X. Yu *et al.*, "Epidemiological and clinical characteristics of 333 confirmed cases with coronavirus disease 2019 in Shanghai, China," *Transbound Emerg Dis*, vol. 67, no. 4, pp. 1697–1707, Jul. 2020, doi: 10.1111/tbed.13604.

[11] S. Yu *et al.*, "Epidemiological Characteristics and Transmissibility for SARS-CoV-2 of Population Level and Cluster Level in a Chinese City," *Front. Public Health*, vol. 9, p. 799536, Jan. 2022, doi: 10.3389/fpubh.2021.799536.

[12] C. You *et al.*, "Estimation of the time-varying reproduction number of COVID-19 outbreak in China," *International Journal of Hygiene and Environmental Health*, vol. 228, p. 113555, Jul. 2020, doi: 10.1016/j.ijheh.2020.113555.

[13] N. Yang *et al.*, "In-flight Transmission Cluster of COVID-19: A Retrospective Case Series".

[14] J. Yang *et al.*, "Clinical characteristics, treatment, and prognosis of 74 2019 novel coronavirus disease patients in Hefei: A single-center retrospective study," *Medicine*, vol. 100, no. 21, p. e25645, May 2021, doi: 10.1097/MD.0000000000025645.

[15] X.-W. Xu *et al.*, "Clinical findings in a group of patients infected with the 2019 novel

coronavirus (SARS-Cov-2) outside of Wuhan, China: retrospective case series," *BMJ*, p. m606, Feb. 2020, doi: 10.1136/bmj.m606.
[16] T. Xu *et al.*, "Clinical features and dynamics of viral load in imported and non-imported patients with COVID-19," *International Journal of Infectious Diseases*, vol. 94, pp. 68–71, May 2020, doi: 10.1016/j.ijid.2020.03.022.
[17] H. Xin *et al.*, "Estimating the Latent Period of Coronavirus Disease 2019 (COVID-19)," *Clinical Infectious Diseases*, vol. 74, no. 9, pp. 1678–1681, May 2022, doi: 10.1093/cid/ciab746.
[18] S. Xie *et al.*, "The epidemiologic and clinical features of suspected and confirmed cases of imported 2019 novel coronavirus pneumonia in north Shanghai, China," *Ann Transl Med*, vol. 8, no. 10, pp. 637–637, May 2020, doi: 10.21037/atm-20-2119.
[19] Z. Xiao *et al.*, "Examining the incubation period distributions of COVID-19 on Chinese patients with different travel histories," *J Infect Dev Ctries*, vol. 14, no. 04, pp. 323–327, Apr. 2020, doi: 10.3855/jidc.12718.
[20] Z. Xiao, W. Guo, Z. Luo, J. Liao, F. Wen, and Y. Lin, "Examining geographical disparities in the incubation period of the COVID-19 infected cases in Shenzhen and Hefei, China," *Environ Health Prev Med*, vol. 26, no. 1, p. 10, Dec. 2021, doi: 10.1186/s12199-021-00935-3.
[21] F. Xiao, B. Chen, T. Xiao, S. K. Lee, K. Yan, and L. Hu, "Children with SARS-CoV-2 infection during an epidemic in China (outside of Hubei province)," *Ann Transl Med*, vol. 8, no. 14, pp. 849–849, Jul. 2020, doi: 10.21037/atm-20-2908.
[22] J. Wu *et al.*, "Epidemiological and clinical features of SARS-CoV-2 cluster infection in Anhui Province, Eastern China," *International Journal of Infectious Diseases*, vol. 117, pp. 372–377, Apr. 2022, doi: 10.1016/j.ijid.2021.04.064.
[23] J. Wong *et al.*, "Epidemiological Investigation of the First 135 COVID-19 Cases in Brunei: Implications for Surveillance, Control, and Travel Restrictions," *The American Journal of Tropical Medicine and Hygiene*, vol. 103, no. 4, pp. 1608–1613, Oct. 2020, doi: 10.4269/ajtmh.20-0771.
[24] Y. S. Won, J.-H. Kim, C. Y. Ahn, and H. Lee, "Subcritical Transmission in the Early Stage of COVID-19 in Korea," *IJERPH*, vol. 18, no. 3, p. 1265, Jan. 2021, doi: 10.3390/ijerph18031265.
[25] Y. Wei *et al.*, "Clinical characteristics of 276 hospitalized patients with coronavirus disease 2019 in Zengdu District, Hubei Province: a single-center descriptive study," *BMC Infect Dis*, vol. 20, no. 1, p. 549, Dec. 2020, doi: 10.1186/s12879-020-05252-8.
[26] Y. Wang *et al.*, "Clinical Characteristics of Patients Infected With the Novel 2019 Coronavirus (SARS-Cov-2) in Guangzhou, China," *Open Forum Infectious Diseases*, vol. 7, no. 6, p. ofaa187, Jun. 2020, doi: 10.1093/ofid/ofaa187.
[27] X. Wang *et al.*, "Nosocomial outbreak of COVID-19 pneumonia in Wuhan, China," *Eur Respir J*, vol. 55, no. 6, p. 2000544, Jun. 2020, doi: 10.1183/13993003.00544-2020.
[28] V. Viego, M. Geri, J. Castiglia, and E. Jouglard, "Incubation period and serial interval of Covid-19 in a chain of infections in Bahia Blanca (Argentina)," Jun. 20, 2020. doi: 10.1101/2020.06.18.20134825.
[29] S. Tian *et al.*, "Characteristics of COVID-19 infection in Beijing," *Journal of Infection*, vol. 80, no. 4, pp. 401–406, Apr. 2020, doi: 10.1016/j.jinf.2020.02.018.
[30] The SARS-CoV-2 variant with lineage B.1.351 clusters investigation team, "Linked transmission chains of imported SARS-CoV-2 variant B.1.351 across mainland France,

January 2021," *Eurosurveillance*, vol. 26, no. 13, Apr. 2021, doi: 10.2807/1560-7917.ES.2021.26.13.2100333.

[31] L. Testing Team, W. Zhang, and Shunyi District Center for Disease Control and Prevention, Beijing, China, "Local Outbreak of COVID-19 in Shunyi District Attributed to an Asymptomatic Carrier with a History of Stay in Indonesia — Beijing Municipality, China, December 23, 2020," *China CDC Weekly*, vol. 3, no. 10, pp. 214–217, 2021, doi: 10.46234/ccdcw2020.062.

[32] H. Tanaka *et al.*, "Shorter Incubation Period among COVID-19 Cases with the BA.1 Omicron Variant," *IJERPH*, vol. 19, no. 10, p. 6330, May 2022, doi: 10.3390/ijerph19106330.

[33] W. Y. T. Tan, L. Y. Wong, Y. S. Leo, and M. P. H. S. Toh, "Does incubation period of COVID-19 vary with age? A study of epidemiologically linked cases in Singapore," *Epidemiol. Infect.*, vol. 148, p. e197, 2020, doi: 10.1017/S0950268820001995.

[34] N. Sugano, W. Ando, and W. Fukushima, "Cluster of Severe Acute Respiratory Syndrome Coronavirus 2 Infections Linked to Music Clubs in Osaka, Japan," *The Journal of Infectious Diseases*, vol. 222, no. 10, pp. 1635–1640, Oct. 2020, doi: 10.1093/infdis/jiaa542.

[35] Y.-J. Su, K.-C. Kuo, T.-W. Wang, and C.-W. Chang, "Gender-based differences in COVID-19," *New Microbes and New Infections*, vol. 42, p. 100905, Jul. 2021, doi: 10.1016/j.nmni.2021.100905.

[36] Y. S. Song, Y. B. Hao, W. W. Liu, S. S. Zhang, P. Wang, and T. L. Fan, "Clinical features of 17 patients with 2019-nCoV," *Eur Rev Med Pharmacol Sci*, vol. 24, pp. 10896–10901, 2020.

[37] E. Shiel *et al.*, "Clinical characteristics and outcomes of COVID -19 in a low-prevalence, well resourced setting, Sydney, Australia," *Internal Medicine Journal*, vol. 51, no. 10, pp. 1605–1613, Oct. 2021, doi: 10.1111/imj.15445.

[38] P. Shi *et al.*, "Characteristics and evaluation of the effectiveness of monitoring and control measures for the first 69 Patients with COVID-19 from 18 January 2020 to 2 March in Wuxi, China," *Sustainable Cities and Society*, vol. 64, p. 102559, Jan. 2021, doi: 10.1016/j.scs.2020.102559.

[39] J.-C. Shi *et al.*, "Epidemiological Features of 105 Patients Infected with the COVID-19," *Journal of the National Medical Association*, vol. 113, no. 2, pp. 212–217, Apr. 2021, doi: 10.1016/j.jnma.2020.09.151.

[40] Y. Shen *et al.*, "A Cluster of Novel Coronavirus Disease 2019 Infections Indicating Person-to-Person Transmission Among Casual Contacts From Social Gatherings: An Outbreak Case-Contact Investigation," *Open Forum Infectious Diseases*, vol. 7, no. 6, p. ofaa231, Jun. 2020, doi: 10.1093/ofid/ofaa231.

[41] S. Sanche, Y. T. Lin, C. Xu, E. Romero-Severson, N. Hengartner, and R. Ke, "High Contagiousness and Rapid Spread of Severe Acute Respiratory Syndrome Coronavirus 2," *Emerg. Infect. Dis.*, vol. 26, no. 7, pp. 1470–1477, Jul. 2020, doi: 10.3201/eid2607.200282.

[42] S. M. Samrah *et al.*, "Viral Clearance Course of COVID-19 Outbreaks," *JMDH*, vol. Volume 14, pp. 555–565, Mar. 2021, doi: 10.2147/JMDH.S302891.

[43] X. Ren *et al.*, "Evidence for pre-symptomatic transmission of coronavirus disease 2019 (COVID-19) in China," *Influenza Resp Viruses*, vol. 15, no. 1, pp. 19–26, Jan. 2021, doi: 10.1111/irv.12787.

[44] R. Ratovoson *et al.*, "Household transmission of COVID-19 among the earliest cases in

Antananarivo, Madagascar," *Influenza Resp Viruses*, vol. 16, no. 1, pp. 48–55, Jan. 2022, doi: 10.1111/irv.12896.
[45] C. Qiu *et al.*, "Transmission and clinical characteristics of coronavirus disease 2019 in 104 outside-Wuhan patients, China," *Journal of Medical Virology*, vol. 92, no. 10, pp. 2027–2035, Oct. 2020, doi: 10.1002/jmv.25975.
[46] G.-Q. Qian *et al.*, "Epidemiologic and clinical characteristics of 91 hospitalized patients with COVID-19 in Zhejiang, China: a retrospective, multi-centre case series," *QJM: An International Journal of Medicine*, vol. 113, no. 7, pp. 474–481, Jul. 2020, doi: 10.1093/qjmed/hcaa089.
[47] B. Qi *et al.*, "Protecting Healthcare Professionals during the COVID-19 Pandemic," *BioMed Research International*, vol. 2020, no. 1, p. 8469560, Jan. 2020, doi: 10.1155/2020/8469560.
[48] R. Pung *et al.*, "Investigation of three clusters of COVID-19 in Singapore: implications for surveillance and response measures," *The Lancet*, vol. 395, no. 10229, pp. 1039–1046, Mar. 2020, doi: 10.1016/S0140-6736(20)30528-6.
[49] K. Ping *et al.*, "Epidemiologic Characteristics of COVID-19 in Guizhou Province, China," *J Infect Dev Ctries*, vol. 15, no. 03, pp. 389–397, Mar. 2021, doi: 10.3855/jidc.12818.
[50] S. Paul and E. Lorin, "Distribution of incubation periods of COVID-19 in the Canadian context," *Sci Rep*, vol. 11, no. 1, p. 12569, Jun. 2021, doi: 10.1038/s41598-021-91834-8.
[51] S. Patrikar *et al.*, "Incubation Period and Reproduction Number for novel coronavirus (COVID-19) infections in India".
[52] D. Pak, K. Langohr, J. Ning, J. Cortés Martínez, G. Gómez Melis, and Y. Shen, "Modeling the Coronavirus Disease 2019 Incubation Period: Impact on Quarantine Policy," *Mathematics*, vol. 8, no. 9, p. 1631, Sep. 2020, doi: 10.3390/math8091631.
[53] T. Ogata, H. Tanaka, F. Irie, A. Hirayama, and Y. Takahashi, "Shorter Incubation Period among Unvaccinated Delta Variant Coronavirus Disease 2019 Patients in Japan," *IJERPH*, vol. 19, no. 3, p. 1127, Jan. 2022, doi: 10.3390/ijerph19031127.
[54] X. Nie *et al.*, "Epidemiological Characteristics and Incubation Period of 7015 Confirmed Cases With Coronavirus Disease 2019 Outside Hubei Province in China," *The Journal of Infectious Diseases*, vol. 222, no. 1, pp. 26–33, Jun. 2020, doi: 10.1093/infdis/jiaa211.
[55] T.-C. Ng *et al.*, "Comparison of Estimated Effectiveness of Case-Based and Population-Based Interventions on COVID-19 Containment in Taiwan," *JAMA Intern Med*, vol. 181, no. 7, p. 913, Jul. 2021, doi: 10.1001/jamainternmed.2021.1644.
[56] B. Moazzami and K. Samimi, "Is Computed Tomography Necessary for the Diagnosis of Coronavirus Disease (COVID–19) in all Suspected Patients? A case series," vol. 28, no. 2, 2021.
[57] P. Llaque-Quiroz, R. Prudencio-Gamio, S. Echevarría-Lo4pez, M. Ccorahua-Paz, and C. Ugas-Charcape, "Características clínicas y epidemiológicas de niños con COVID-19 en un hospital pediátrico del Perú," *Rev Peru Med Exp Salud Publica*, vol. 37, no. 4, pp. 689–93, Nov. 2020, doi: 10.17843/rpmesp.2020.374.6198.
[58] Y. Liu *et al.*, "Comparison of clinical characteristics between patients with coronavirus disease 2019 (COVID-19) who retested RT-PCR positive versus negative: a retrospective study of data from Nanjing," *J Thorac Dis*, vol. 12, no. 11, pp. 6435–6445, Nov. 2020, doi: 10.21037/jtd.2020.04.17.
[59] J.-Y. Liu, T.-J. Chen, and S.-J. Hwang, "Analysis of community-acquired COVID-19 cases in Taiwan," *Journal of the Chinese Medical Association*, vol. 83, no. 12, pp. 1087–1092,

Dec. 2020, doi: 10.1097/JCMA.0000000000000411.
[60] J. Liu *et al.*, “Community Transmission of Severe Acute Respiratory Syndrome Coronavirus 2, Shenzhen, China, 2020,” *Emerg. Infect. Dis.*, vol. 26, no. 6, Jun. 2020, doi: 10.3201/eid2606.200239.
[61] F. Liu *et al.*, “Clinical characteristics and corticosteroids application of different clinical types in patients with corona virus disease 2019,” *Sci Rep*, vol. 10, no. 1, p. 13689, Aug. 2020, doi: 10.1038/s41598-020-70387-2.
[62] N. Linton *et al.*, “Incubation Period and Other Epidemiological Characteristics of 2019 Novel Coronavirus Infections with Right Truncation: A Statistical Analysis of Publicly Available Case Data,” *JCM*, vol. 9, no. 2, p. 538, Feb. 2020, doi: 10.3390/jcm9020538.
[63] Q. Li *et al.*, “Early Transmission Dynamics in Wuhan, China, of Novel Coronavirus–Infected Pneumonia,” *N Engl J Med*, vol. 382, no. 13, pp. 1199–1207, Mar. 2020, doi: 10.1056/NEJMoa2001316.
[64] J. Li *et al.*, “Epidemiological and clinical characteristics of three family clusters of COVID-19 transmitted by latent patients in China,” *Epidemiol. Infect.*, vol. 148, p. e137, 2020, doi: 10.1017/S0950268820001491.
[65] J. Li *et al.*, “Incubation Period of Coronavirus Disease 2019: New Implications for Intervention and Control,” Jan. 18, 2021. doi: 10.21203/rs.3.rs-137093/v1.
[66] C. Leung, “The difference in the incubation period of 2019 novel coronavirus (SARS-CoV-2) infection between travelers to Hubei and nontravelers: The need for a longer quarantine period,” *Infect. Control Hosp. Epidemiol.*, vol. 41, no. 5, pp. 594–596, May 2020, doi: 10.1017/ice.2020.81.
[67] J. J. Lee *et al.*, “Importation and Transmission of SARS-CoV-2 B.1.1.529 (Omicron) Variant of Concern in Korea, November 2021,” *J Korean Med Sci*, vol. 36, no. 50, p. e346, 2021, doi: 10.3346/jkms.2021.36.e346.
[68] T. Q. M. Le *et al.*, “Severe Acute Respiratory Syndrome Coronavirus 2 Shedding by Travelers, Vietnam, 2020,” *Emerg. Infect. Dis.*, vol. 26, no. 7, pp. 1624–1626, Jul. 2020, doi: 10.3201/eid2607.200591.
[69] Y. C. Lau *et al.*, “Joint Estimation of Generation Time and Incubation Period for Coronavirus Disease 2019,” *The Journal of Infectious Diseases*, p. jiab424, Aug. 2021, doi: 10.1093/infdis/jiab424.
[70] C. Lai *et al.*, “Shorter incubation period is associated with severe disease progression in patients with COVID-19,” *Virulence*, vol. 11, no. 1, pp. 1443–1452, Dec. 2020, doi: 10.1080/21505594.2020.1836894.
[71] W. Kong, Y. Wang, J. Hu, A. Chughtai, and H. Pu, “Comparison of clinical and epidemiological characteristics of asymptomatic and symptomatic SARS-CoV-2 infection: A multi-center study in Sichuan Province, China,” *Travel Medicine and Infectious Disease*, vol. 37, p. 101754, Sep. 2020, doi: 10.1016/j.tmaid.2020.101754.
[72] T. Kong, “Longer incubation period of coronavirus disease 2019 (COVID-19) in older adults,” *Aging Medicine*, vol. 3, no. 2, pp. 102–109, Jun. 2020, doi: 10.1002/agm2.12114.
[73] D. Kong *et al.*, “Pre-symptomatic transmission of novel coronavirus in community settings,” *Influenza Resp Viruses*, vol. 14, no. 6, pp. 610–614, Nov. 2020, doi: 10.1111/irv.12773.
[74] K. Khonyongwa *et al.*, “Incidence and outcomes of healthcare-associated COVID-19 infections: significance of delayed diagnosis and correlation with staff absence,” *Journal of Hospital Infection*, vol. 106, no. 4, pp. 663–672, Dec. 2020, doi:

10.1016/j.jhin.2020.10.006.
[75] X. Jin *et al.*, “Epidemiological, clinical and virological characteristics of 74 cases of coronavirus-infected disease 2019 (COVID-19) with gastrointestinal symptoms,” *Gut*, vol. 69, no. 6, pp. 1002–1009, Jun. 2020, doi: 10.1136/gutjnl-2020-320926.
[76] G. Jiang *et al.*, “Aerosol transmission, an indispensable route of COVID-19 spread: case study of a department-store cluster,” *Front. Environ. Sci. Eng.*, vol. 15, no. 3, p. 46, Jun. 2021, doi: 10.1007/s11783-021-1386-6.
[77] T. H. Jeong, C. Pak, M. Ock, S.-H. Lee, J. S. Son, and Y.-J. Jeon, “Real Asymptomatic SARS-CoV-2 Infection Might be Rare: the Importance of Careful Interviews and Follow-up,” *J Korean Med Sci*, vol. 35, no. 37, p. e333, 2020, doi: 10.3346/jkms.2020.35.e333.
[78] D. Je *et al.*, “Demographics, clinical characteristics and outcomes among 197 patients with COVID -19 in the Gold Coast area,” *Internal Medicine Journal*, vol. 51, no. 5, pp. 666–672, May 2021, doi: 10.1111/imj.15260.
[79] C. Hua *et al.*, “Epidemiological features and viral shedding in children with SARS-CoV-2 infection,” *Journal of Medical Virology*, vol. 92, no. 11, pp. 2804–2812, Nov. 2020, doi: 10.1002/jmv.26180.
[80] Y. Han *et al.*, “A comparative-descriptive analysis of clinical characteristics in 2019-coronavirus-infected children and adults,” *Journal of Medical Virology*, vol. 92, no. 9, pp. 1596–1602, Sep. 2020, doi: 10.1002/jmv.25835.
[81] T. Han, “Outbreak investigation: transmission of COVID-19 starting from a spa facility in a local community in Korea,” *Epidemiol Health*, p. e2020056, Jul. 2020, doi: 10.4178/epih.e2020056.
[82] C.-X. Guo *et al.*, “Epidemiological and clinical features of pediatric COVID-19,” *BMC Med*, vol. 18, no. 1, p. 250, Dec. 2020, doi: 10.1186/s12916-020-01719-2.
[83] Y. Gao *et al.*, “Epidemiological and clinical differences of coronavirus disease 2019 patients with distinct viral exposure history,” *Virulence*, vol. 11, no. 1, pp. 1015–1023, Dec. 2020, doi: 10.1080/21505594.2020.1802870.
[84] F. Denis, A.-L. Septans, F. Le Goff, S. Jeanneau, and F.-X. Lescure, “Analysis of COVID-19 Transmission Sources in France by Self-Assessment Before and After the Partial Lockdown: Observational Study,” *J Med Internet Res*, vol. 23, no. 5, p. e26932, May 2021, doi: 10.2196/26932.
[85] L. Deng and Z. Li, “Epidemiological and clinical findings of discharge patients infected with the 2019 novel coronavirus (SARS-CoV-2) in Changchun, Northeast China: a retrospective cohort study,” *Acta Medica Mediterranea*, no. 2, pp. 1147–1153, Apr. 2021, doi: 10.19193/0393-6384_2021_2_176.
[86] J. Del Águila-Mejía, R. Wallmann, J. Calvo-Montes, J. Rodríguez-Lozano, T. Valle-Madrazo, and A. Aginagalde-Llorente, “Secondary Attack Rate, Transmission and Incubation Periods, and Serial Interval of SARS-CoV-2 Omicron Variant, Spain,” *Emerg. Infect. Dis.*, vol. 28, no. 6, pp. 1224–1228, Jun. 2022, doi: 10.3201/eid2806.220158.
[87] F. De Laval *et al.*, “Lessons learned from the investigation of a COVID-19 cluster in Creil, France: effectiveness of targeting symptomatic cases and conducting contact tracing around them,” *BMC Infect Dis*, vol. 21, no. 1, p. 457, Dec. 2021, doi: 10.1186/s12879-021-06166-9.
[88] J. Dai, L. Yang, and J. Zhao, “Probable Longer Incubation Period for Elderly COVID-19 Cases: Analysis of 180 Contact Tracing Data in Hubei Province, China,” *RMHP*, vol.

Volume 13, pp. 1111–1117, Aug. 2020, doi: 10.2147/RMHP.S257907.
[89] G. Chen *et al.*, “Epidemiological analysis of 18 patients with COVID-19,” *Eur Rev Med Pharmacol Sci*, vol. 24, pp. 12522–12526, 2020.
[90] L. T. Brandal *et al.*, “Outbreak caused by the SARS-CoV-2 Omicron variant in Norway, November to December 2021,” *Eurosurveillance*, vol. 26, no. 50, Dec. 2021, doi: 10.2807/1560-7917.ES.2021.26.50.2101147.
[91] C. Bao *et al.*, “COVID-19 outbreak following a single patient exposure at an entertainment site: An epidemiological study,” *Transbounding Emerging Dis*, vol. 68, no. 2, pp. 773–781, Mar. 2021, doi: 10.1111/tbed.13742.
[92] J. A. Backer, D. Klinkenberg, and J. Wallinga, “Incubation period of 2019 novel coronavirus (2019-nCoV) infections among travellers from Wuhan, China, 20–28 January 2020,” *Eurosurveillance*, vol. 25, no. 5, Feb. 2020, doi: 10.2807/1560-7917.ES.2020.25.5.2000062.
[93] J. A. Backer *et al.*, “Shorter serial intervals in SARS-CoV-2 cases with Omicron BA.1 variant compared with Delta variant, the Netherlands, 13 to 26 December 2021,” *Eurosurveillance*, vol. 27, no. 6, Feb. 2022, doi: 10.2807/1560-7917.ES.2022.27.6.2200042.
[94] B. Areekal *et al.*, “Risk Factors, Epidemiological and Clinical Outcome of Close Contacts of COVID-19 Cases in a Tertiary Hospital in Southern India,” *JCDR*, 2021, doi: 10.7860/JCDR/2021/48059.14664.
