# Supplementary material for: Using artificial intelligence tools to automate data extraction for living evidence syntheses
Source: PLoS One. 2025 Apr 3;20(4):e0320151. doi: 10.1371/journal.pone.0320151 (PMC11967977; doi:10.1371/journal.pone.0320151)
Supplement: S3 Appendix — (PDF) [file pone.0320151.s003.pdf]

Supporting information to: Using artificial intelligence tools to automate data extraction for living evidence syntheses

Evan Mitchell, Elisha B. Are, Caroline Colijn, David J. D. Earn

This Supporting Information presents the AI-LES Python script described in the main text.

```
##########################################################
# This script, dubbed AI-LES, interfaces with ChatGPT's  #
# engine to extract study results from a set of journal  #
# articles                                               #
##########################################################

# Import required packages

import csv
import glob
import openai
import PyPDF2
import time

# Enter your OpenAI API key

openai.api_key = 'xxxxx'

# Load all pdfs in the desired directory

pdfs = []
for file in glob.glob('test_papers/*.pdf'):
    pdfs.append(file)

# Define a function wrapper to send messages with exponential backoff

def retry_with_exponential_backoff(
    func,
    initial_delay: float = 1,
    exponential_base: float = 2,
    max_retries: int = 10,
    errors: tuple = (openai.error.RateLimitError,),
):
    """Retry a function with exponential backoff."""

    def wrapper(*args, **kwargs):
        # Initialize variables
```

```python
        num_retries = 0
        delay = initial_delay
        total_delay = 0

        # Loop until a successful response or max_retries is hit
        while True:
            try:
                return (func(*args, **kwargs), total_delay)

            # Retry on specified errors
            except errors:
                # Increment retries
                num_retries += 1

                # Check if max retries has been reached
                if num_retries > max_retries:
                    raise Exception(
                        f"Maximum number of retries \
                        ({max_retries}) exceeded."
                    )

                # Increment the delay
                delay *= exponential_base
                total_delay += delay

                # Sleep for the delay
                time.sleep(delay)

    return wrapper


# Attach this wrapper to a function that contacts ChatGPT's engine

@retry_with_exponential_backoff
def completion_with_backoff(**kwargs):
    return openai.ChatCompletion.create(**kwargs)

# Define a function to send multiple queries to ChatGPT

def continue_chat(user_msg):
    # Organize messages and responses as a vector and
    # assign the correct role to each
    allmsgs = [
        {"role": "assistant", "content": user_msg[i]} if i % 2
        else {"role": "user", "content": user_msg[i]}
        for i in range(len(user_msg))
    ]

    # Send the messages to ChatGPT using exponential backoff
    response = completion_with_backoff(model="gpt-3.5-turbo-0125",
                                       temperature = 0,
                                       messages=allmsgs)

    # Return ChatGPT's response to the most recent query,
```

```python
    # as well as the total delay time (in seconds)
    return (response[0].choices[0].message.content, response[1])

# Pass each pdf to ChatGPT

results = []
for article in pdfs:
    print("Working on article:", article)
    responses = []
    responses.append(article)

    # Read in and extract the pdf article text
    pdf_file_obj = open(article, 'rb')
    pdf_reader = PyPDF2.PdfReader(pdf_file_obj)
    num_pages = len(pdf_reader.pages)
    detected_text = ''
    for page_num in range(num_pages):
        page_obj = pdf_reader.pages[page_num]
        detected_text += page_obj.extract_text() + '\n\n'
    pdf_file_obj.close()

    # Calculate start time
    t0 = time.time()
    delay_time = 0

    # Call ChatGPT to interface with the pdf
    query_init = 'What is the estimate for the incubation period from \
    this paper? Please reply with a single number, or NA if \
    not provided.'
    user_msg_init = detected_text + "\n\n" + query_init
    response = completion_with_backoff(model = "gpt-3.5-turbo-0125",
                                       temperature = 0,
                                       messages = [
                                           {"role": "user", "content":
                                           user_msg_init}
                                       ],
    )
    delay_time += response[1]
    response_init = response[0].choices[0].message.content
    responses.append(response_init)
    user_msg = [user_msg_init, response_init]
    query1 = 'Is this an estimate of the mean or median \
    incubation period?'
    query2 = 'What is the 95% confidence interval, range, \
    or IQR for the incubation period? Please reply with two \
    numbers giving the lower and upper bounds, or NA if not provided.'
    query3 = 'Is this a confidence interval, a range, or an IQR?'
    query4 = 'What is the standard deviation or standard error of the \
    incubation period? Please reply with a single number, or NA if \
    not provided.'
    query5 = 'Is this a standard deviation or a standard error?'
    query6 = 'Does this paper present incubation periods for different \
    subgroups of individuals? If no, please return a 0. If yes, please \
    list the subgroups.'
```

```python
    queries = [query1, query2, query3, query4, query5, query6]
    for i in range(len(queries)):
        user_msg.append(queries[i])
        response = continue_chat(user_msg)
        delay_time += response[1]
        responses.append(response[0])
        user_msg.append(response[0])
    print("> completed")

    # Calculate end time
    t1 = time.time()

    # Calculate total runtime
    proc_time = round(t1 - t0 - delay_time, 2)

    # Add results from this article to the results from other articles
    responses.append(proc_time)
    responses.append(delay_time)
    results.append(responses)

print("All articles have been processed")

# Save results to a CSV file

with open('results.csv', 'w', newline = '') as file:
    writer = csv.writer(file)
    writer.writerow(['Article Location',
                     'Mean Incubation Period Estimate',
                     'Mean or Median?',
                     '95% CI', 'CI or Range?',
                     'Standard Deviation',
                     'SD or SE?',
                     'Subgroups Present?',
                     'Processing Time (sec)',
                     'Delay Time (sec)'])
    for i in range(len(results)):
        writer.writerow(results[i])
```
